# Supplementary figures and images for: Exploratory association between multimodal AI-derived digital biomarkers and in-hospital mortality in adult patients with pneumonia: A proof-of-concept study
Source: PLOS Digit Health. 2026 Apr 30;5(4):e0000960. doi: 10.1371/journal.pdig.0000960 (PMC13132438; doi:10.1371/journal.pdig.0000960)

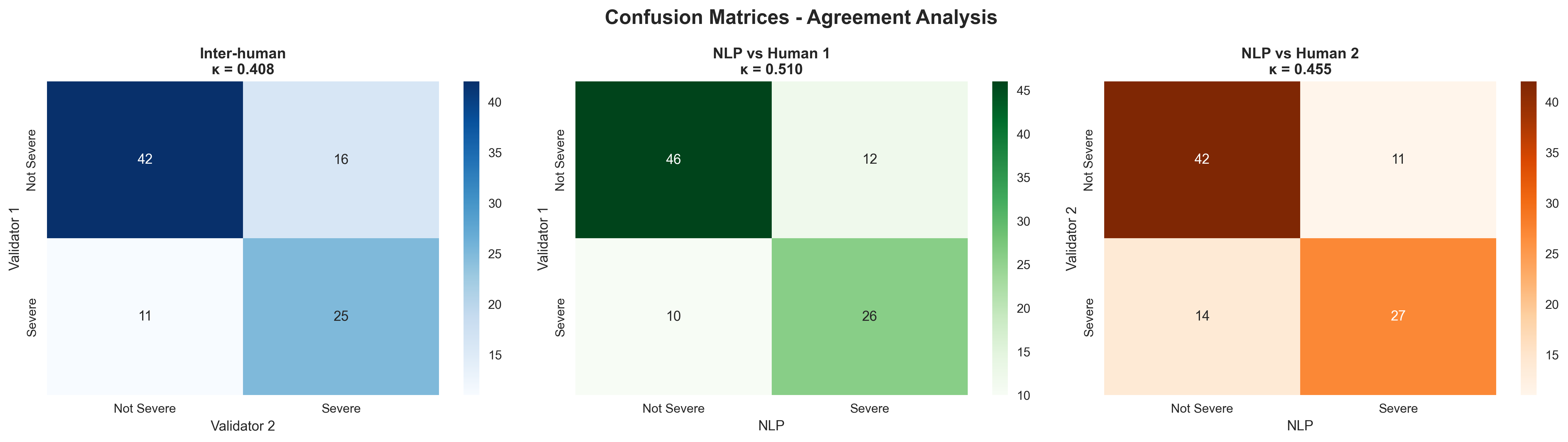

Supplement: S1 Fig — Confusion matrices for each IDSA/ATS criterion comparing NLP-V1 and NLP-V2 predictions against human expert consensus (n = 94 double-annotated records). (TIF) [file pdig.0000960.s001.tif]

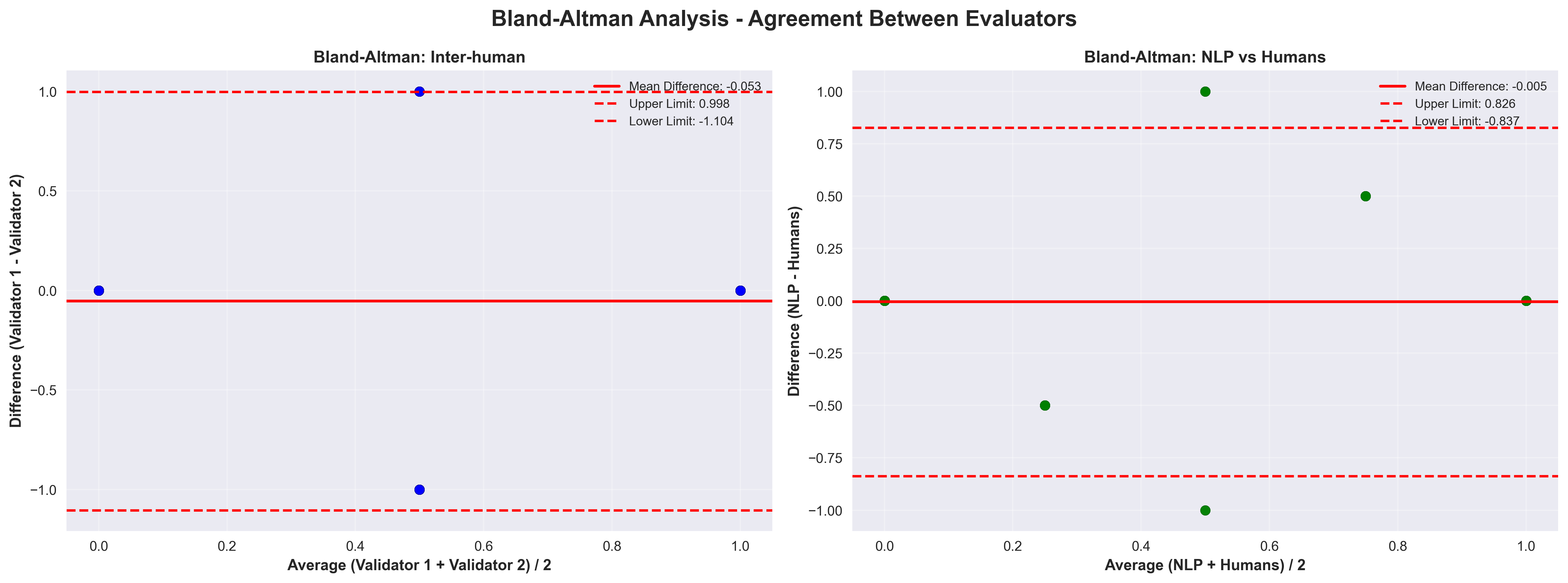

Supplement: S2 Fig — Available digitisation accuracy plots from the PDF ECG pipeline. Note: comparison against native machine-output digital ECG (XML/SCP-ECG) was not possible for this cohort; all ECGs were archived as paper reports only. (TIF) [file pdig.0000960.s002.tif]

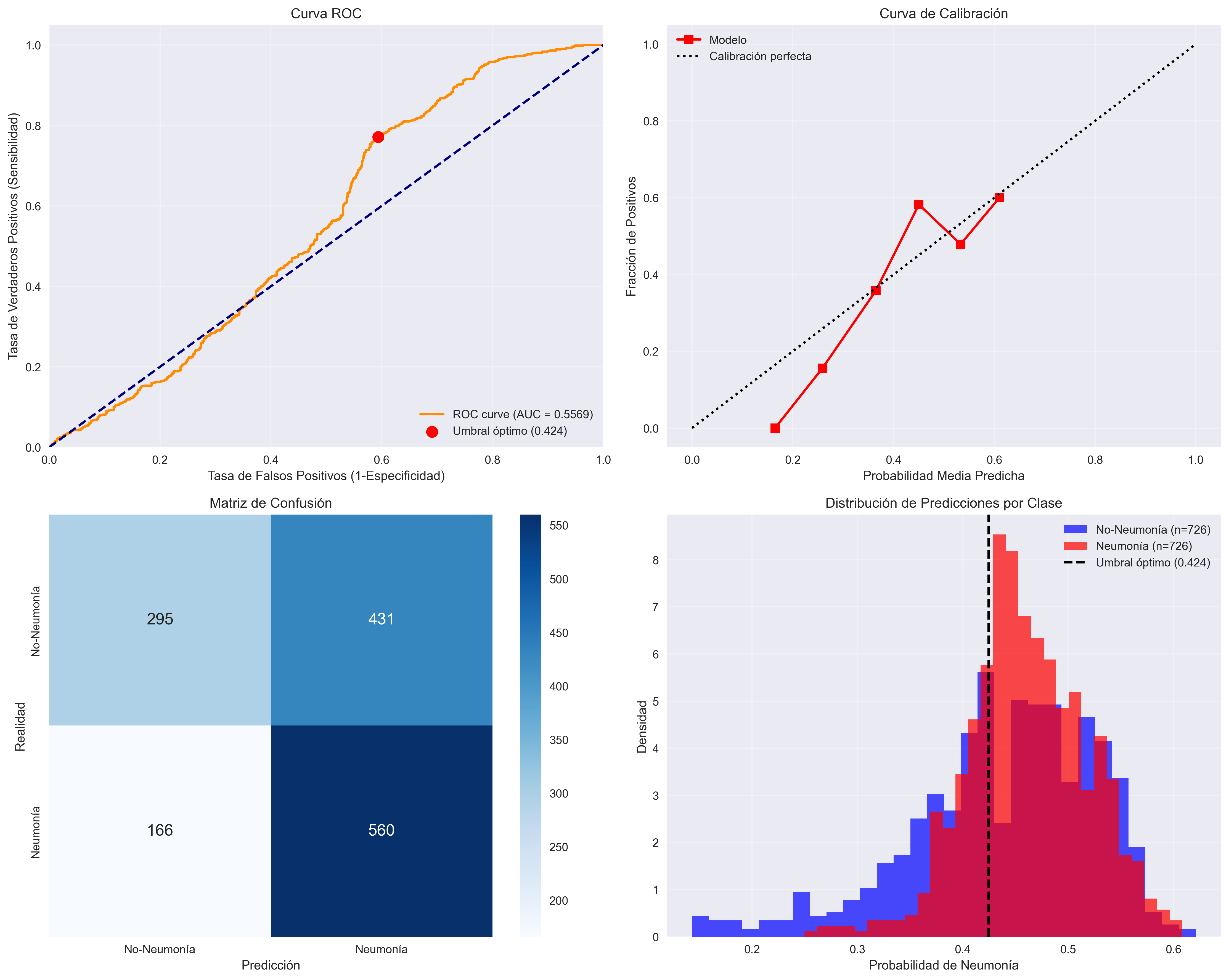

Supplement: S3 Fig — Receiver operating characteristic (ROC) curves for pneumonia vs. healthy (AUC = 0.624) and pneumonia vs. other disease (AUC = 0.556) binary comparisons. Calibration plot shown with Brier score. (TIF) [file pdig.0000960.s003.tif]

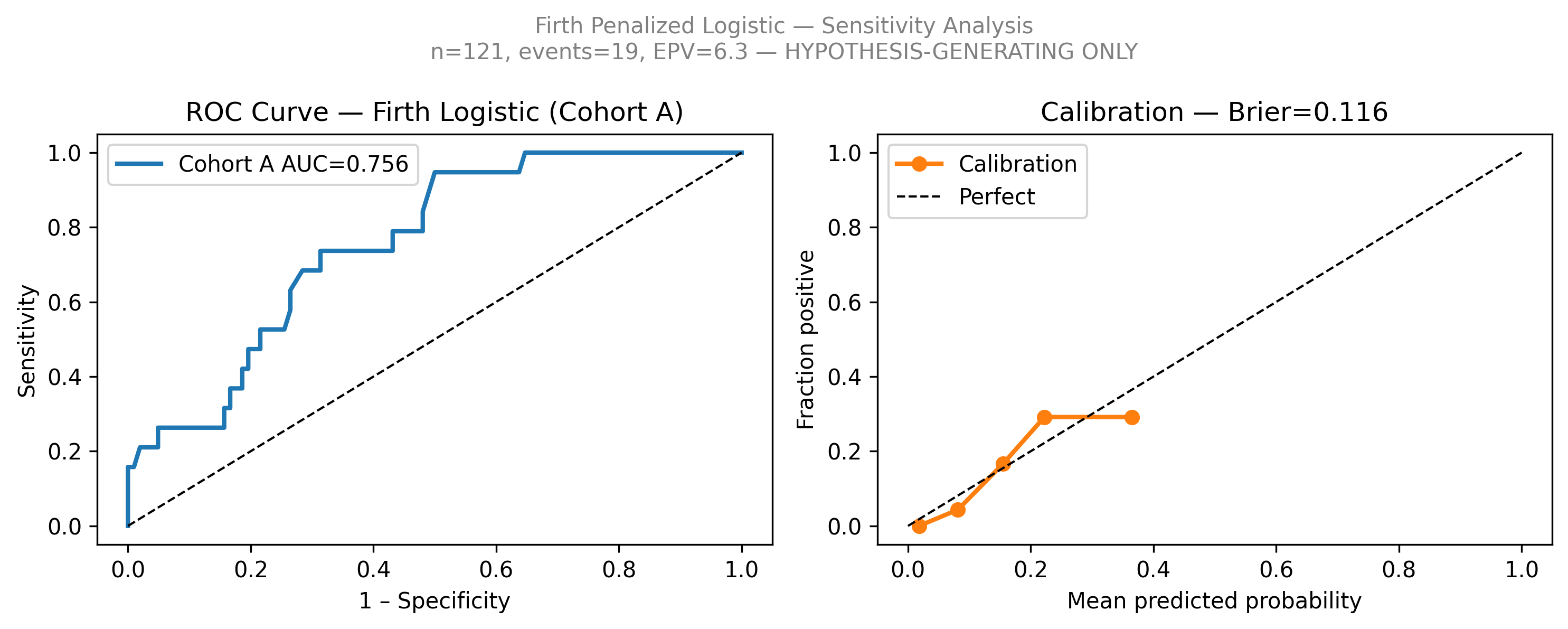

Supplement: S4 Fig — Receiver operating characteristic curve (AUC = 0.756, 95% CI: 0.625–0.887) and calibration plot (Brier score = 0.116) for the Firth penalised logistic model fitted on Cohort A (n = 121) with three predictors (CXR total compromise ratio, NLP-IDSA score, age). See S7 Table for coefficient estimates. (TIF) [file pdig.0000960.s004.tif]

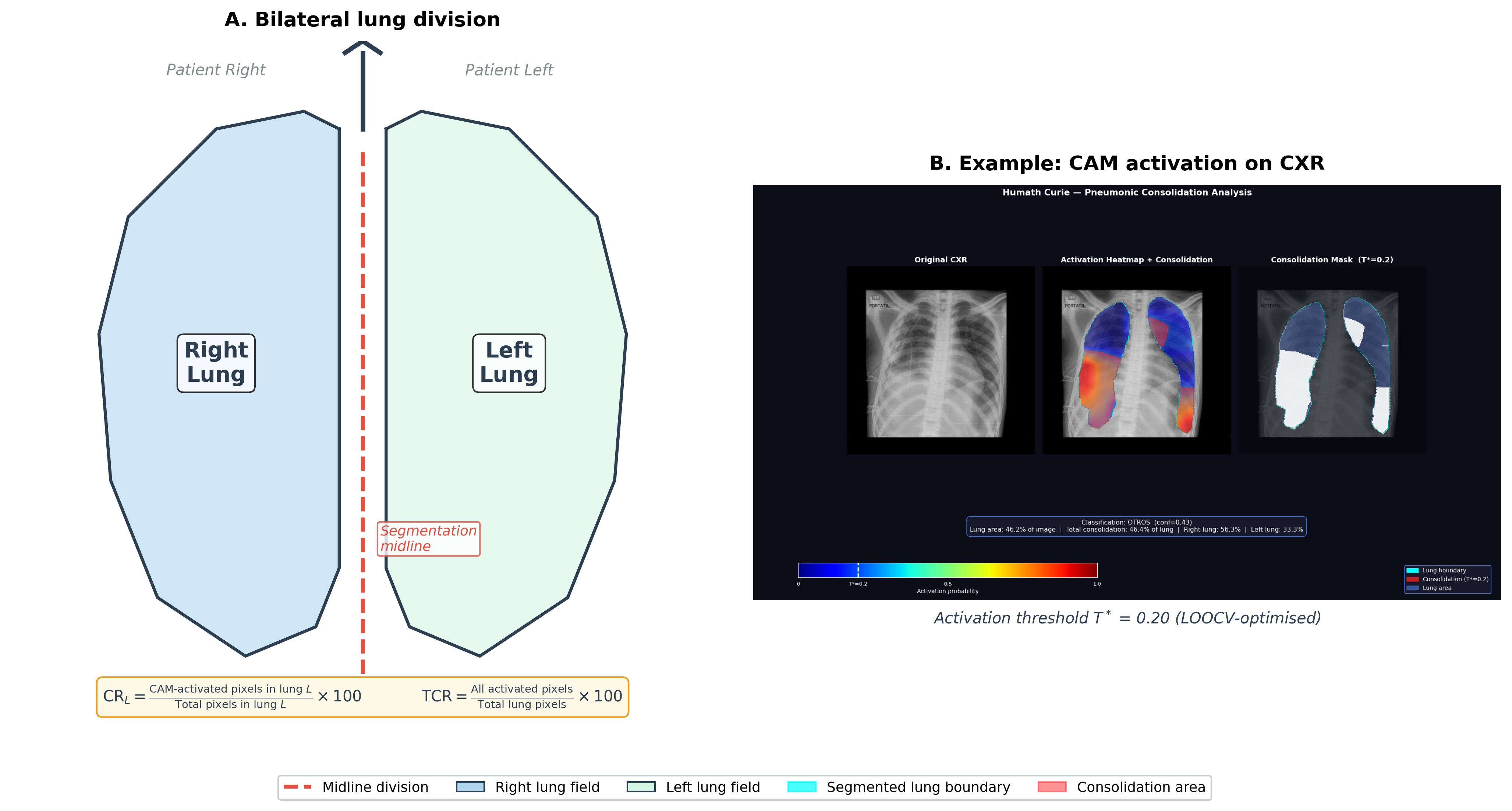

Supplement: S5 Fig — Panel A: Bilateral lung division used to compute the compromise ratio (CRL) for the right and left lungs along the segmentation midline. Panel B: Representative CXR processed through the Humath Curie pipeline showing the original image, Class Activation Map (CAM) heatmap overlay with consolidation area in red, and binary consolidation mask at threshold T* = 0.20. (TIF) [file pdig.0000960.s005.tif]

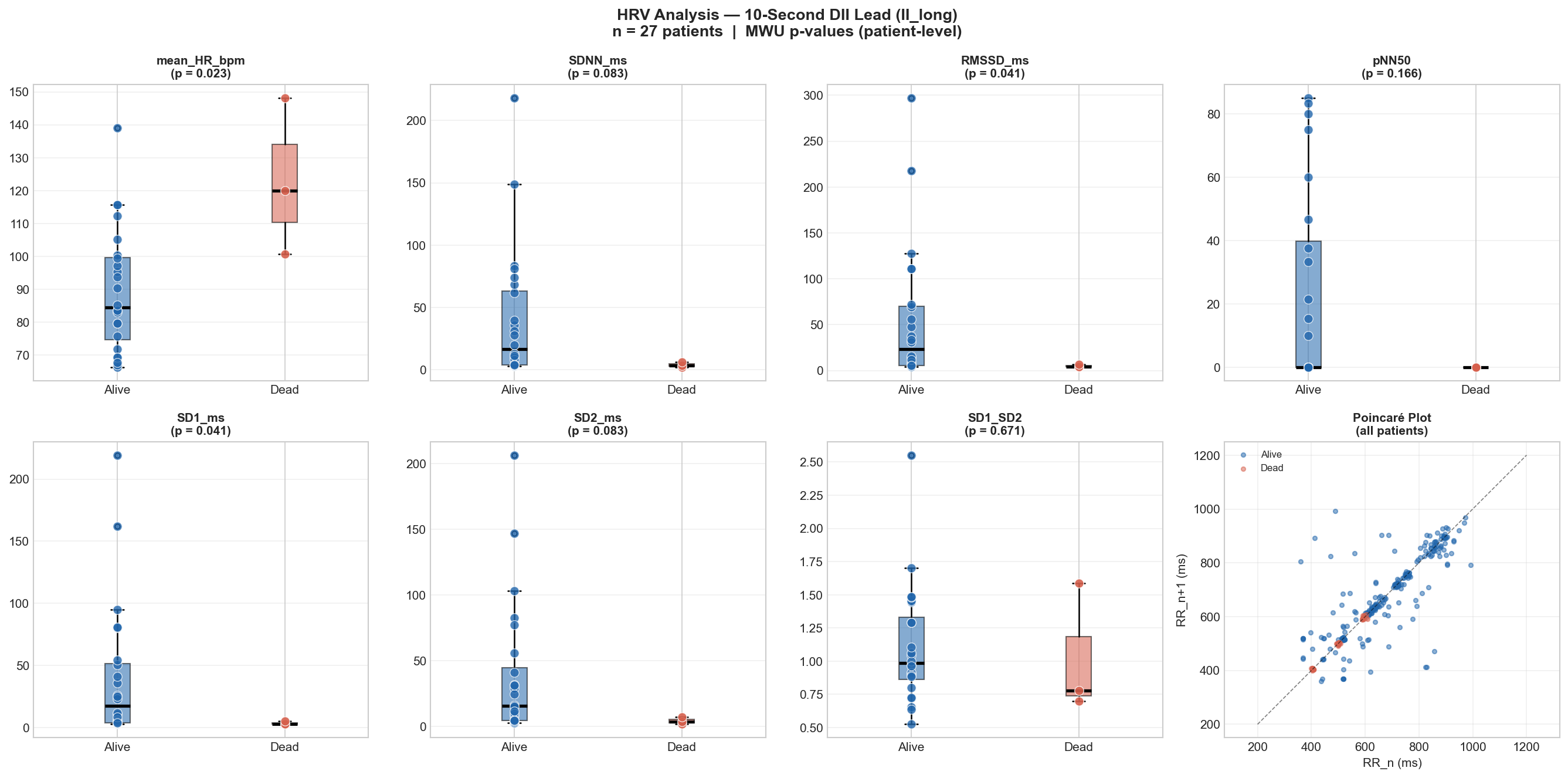

Supplement: S6 Fig — Box plots comparing heart rate variability indices derived from the standardised lead II segment (n = 27 patients: 24 survivors, 3 non-survivors). (TIF) [file pdig.0000960.s006.tif]

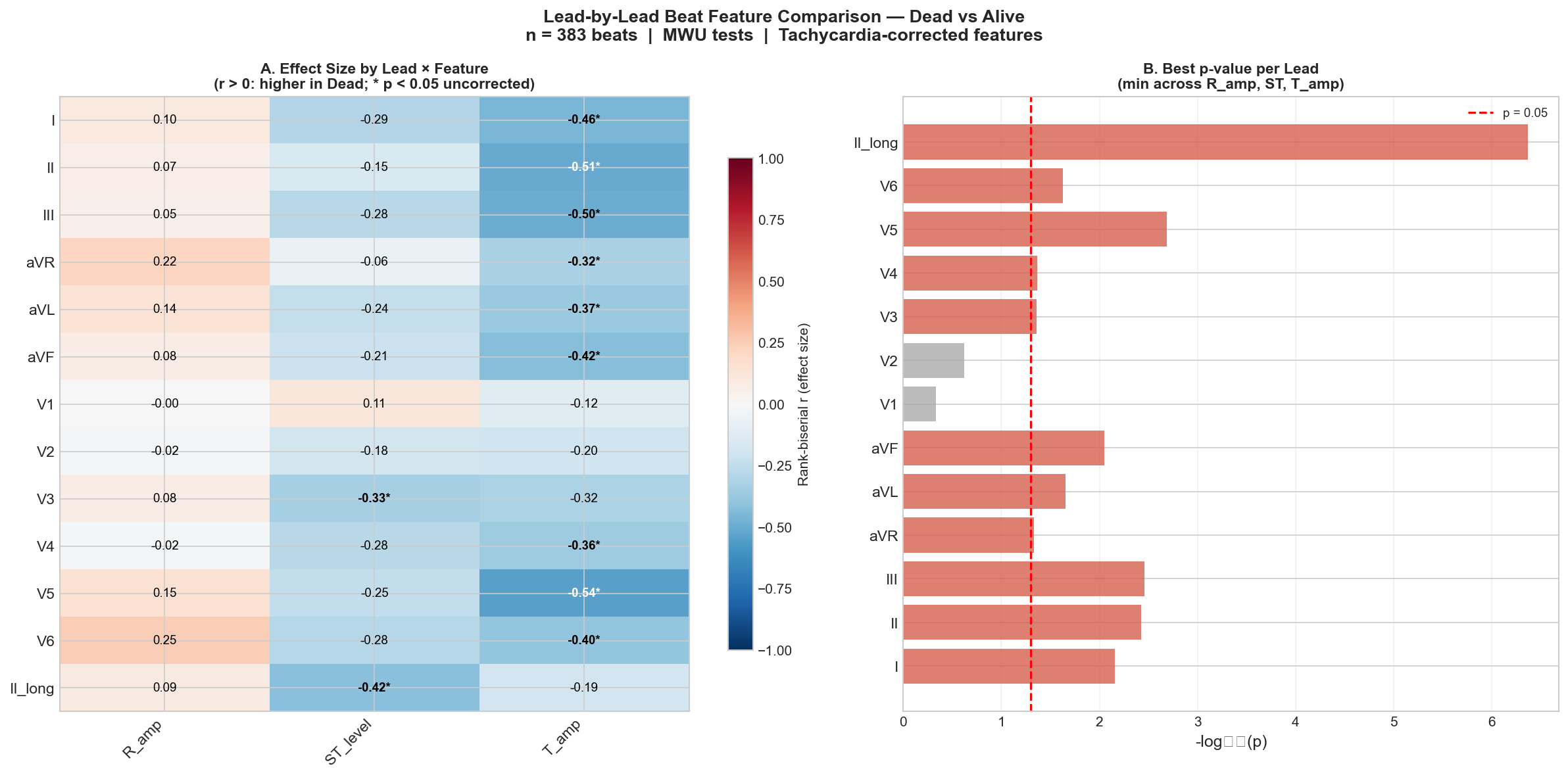

Supplement: S7 Fig — Wilcoxon rank-sum comparisons of R-wave amplitude, ST-segment level, and T-wave amplitude across 13 leads. Four features survived Benjamini-Hochberg FDR correction at q<0.05. (TIF) [file pdig.0000960.s007.tif]

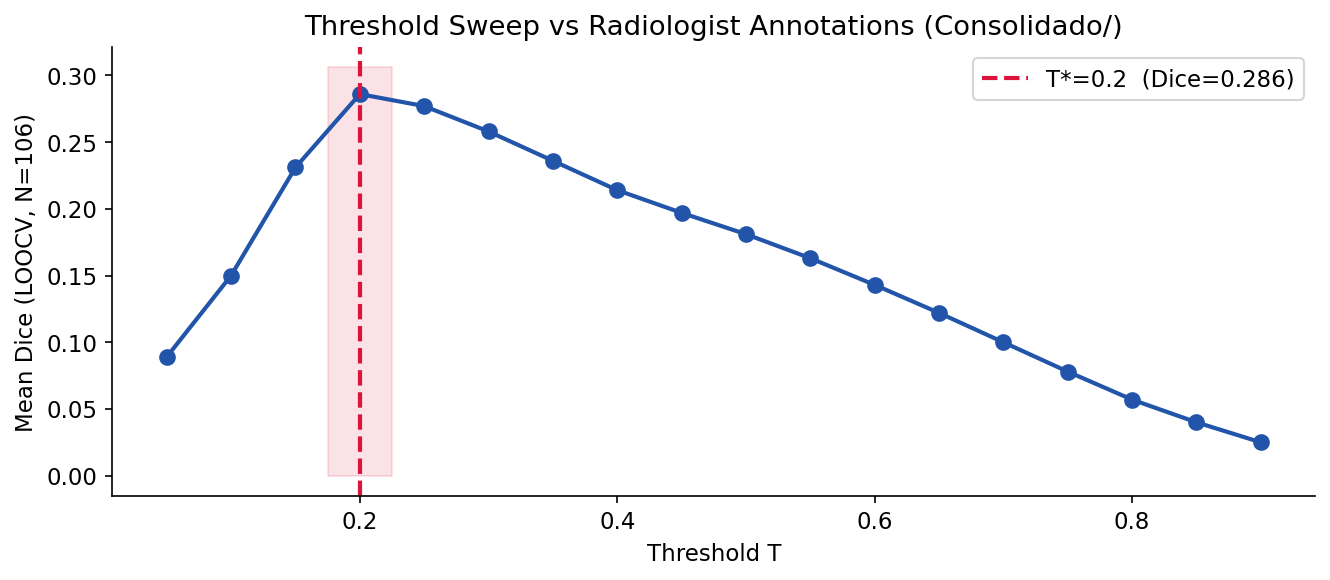

Supplement: S8 Fig — Dice coefficient and AUROC as a function of the CAM activation threshold T across 106 radiologist-annotated images. Optimal threshold T* = 0.20 maximised Dice (0.286±0.199; AUROC = 0.613±0.223). (TIF) [file pdig.0000960.s008.tif]
