# Supplementary material for: Exploratory association between multimodal AI-derived digital biomarkers and in-hospital mortality in adult patients with pneumonia: A proof-of-concept study
Source: PLOS Digit Health. 2026 Apr 30;5(4):e0000960. doi: 10.1371/journal.pdig.0000960 (PMC13132438; doi:10.1371/journal.pdig.0000960)
Supplement: S1 Appendix — Completed STROBE checklist for prospective/retrospective observational studies (cohort design). (PDF) [file pdig.0000960.s018.pdf]

S1 Appendix. STROBE Observational Study Compliance Checklist

The following checklist documents compliance with the Strengthening the Reporting of Observational Studies in Epidemiology (STROBE) statement for cohort studies [1]. Items marked with an asterisk (*) indicate where information is provided for the relevant study design(s).

**Section/Topic**	**Item**	**Recommendation**	**Reported on page / section**
**Title and abstract**			
Title and abstract	1	(a) Indicate the study's design with a commonly used term in the title or the abstract	Title: "exploratory ... proof-of-concept study"; Abstract para. 2: "exploratory, proof-of-concept retrospective study"
		(b) Provide an informative and balanced summary of what was done and what was found	Abstract (full)
**Introduction**			
Background / rationale	2	Explain the scientific background and rationale for the investigation being reported	Introduction, paras. 1–4
Objectives	3	State specific objectives, including any prespecified hypotheses	Introduction, final paragraph ("This exploratory ... aimed to examine associations...")
**Methods**			
Study design	4	Present key elements of study design early in the manuscript	Abstract para. 2; Materials and Methods, Ethics statement
Setting	5	Describe the setting, locations, and relevant dates, including periods of recruitment, exposure, follow-up, and data collection	Materials and Methods, Study participants: "Hospital Alma Máter de Antioquia, Medellín, Colombia; January 1 to June 30, 2024"
Participants	6	(a) Give eligibility criteria, sources and methods of selection of participants. Describe methods of follow-up	Materials and Methods, Study participants: inclusion criteria (age $\geq$18, primary pneumonia diagnosis, continuous monitoring requirement); exclusion criteria (refusal of intubation, >20% missingness)
		(b) For matched studies, give matching criteria and number of exposed and unexposed	N/A — unmatched retrospective cohort

**Section/Topic**	**Item**	**Recommendation**	**Reported on page / section**
Variables	7	Clearly define all outcomes, exposures, predictors, potential confounders, and effect modifiers. Give diagnostic criteria, if applicable	Materials and Methods: outcome = all-cause in-hospital mortality; predictors defined per modality (CXR compromise ratio, NLP severity score, ECG/HRV features). ECG waveform definitions in Results. IDSA/ATS criteria defined in NLP pipeline subsection.
Data sources / measurement	8*	For each variable of interest, give sources of data and details of methods of assessment (measurement). Describe comparability of assessment methods if there is more than one group	Materials and Methods: Data sources and model characteristics; ECG digitisation and signal processing; NLP pipeline; CXR pipeline with CAM
Bias	9	Describe any efforts to address potential sources of bias	Materials and Methods: Ethics statement (intubation exclusion rationale); Study participants (missingness threshold); Limitations (selection, temporal, and geographic bias)
Study size	10	Explain how the study size was arrived at	Study participants: consecutive admissions Jan–Jun 2024; n = 184 screened, n = 121 after exclusions; 19 events. Limitations acknowledges low EPV.
Quantitative variables	11	Explain how quantitative variables were handled in the analyses. If applicable, describe which groupings were chosen and why	Statistical analysis: ORs per SD increase for continuous variables; binary outcome (death yes/no)
Statistical methods	12	(a) Describe all statistical methods, including those used to control for confounding	Statistical analysis: univariate logistic regression, Mann-Whitney U, Fisher's exact, Firth penalised regression (S7 Table)
		(b) Describe any methods used to examine subgroups and interactions	Sex-specific and age-stratified mortality rates in Results; ECG subgroup (n = 27)

Section/Topic	Item	Recommendation	Reported on page / section
		(c) Explain how missing data were addressed	Study participants: >20% missingness exclusion threshold (Sterne et al. 2009); S1 Table reports missingness
		(d) If applicable, explain how loss to follow-up was addressed	N/A — in-hospital outcome; no loss to follow-up
		(e) Describe any sensitivity analyses	Firth penalised logistic regression (S7 Table, S4 Fig); BH-FDR correction for ECG features; spline/non-linear analysis considered but insufficient df
**Results**			
Participants	13*	(a) Report numbers of individuals at each stage of study — e.g., numbers potentially eligible, examined for eligibility, confirmed eligible, included in the study, completing follow-up, and analysed	Results, Cohort characteristics; Fig 1 (STROBE flow diagram)
		(b) Give reasons for non-participation at each stage	Fig 1: age <18 (n = 42); poor-quality CXR (n = 21)
		(c) Consider use of a flow diagram	Fig 1
Descriptive data	14*	(a) Give characteristics of study participants (demographic, clinical) and information on exposures and potential confounders	Table 1: age, sex, LOS, primary diagnosis, antibiotics
		(b) Indicate number of participants with missing data for each variable of interest	S1 Table (missingness by cohort)
		(c) Summarise follow-up time	N/A — cross-sectional in-hospital assessment; LOS reported in Table 1; time-to-death in Results
Outcome data	15*	Report numbers of outcome events or summary measures over time	Results: 19/121 non-survivors (15.7%); time-to-death 12.0 days [IQR 5.0–21.5]
Main results	16	(a) Give unadjusted estimates and, if applicable, confounder-adjusted estimates and their precision (95% CI). Make clear which confounders were adjusted for and why they were included	Results: ORs with 95% CIs from univariate logistic regression (Figs 2, 3); Firth model in S7 Table. All analyses unadjusted (exploratory design).

**Section/Topic**	**Item**	**Recommendation**	**Reported on page / section**
		(b) Report category boundaries when continuous variables were categorised	N/A — continuous variables not categorised; ORs per SD
		(c) If relevant, consider translating estimates of relative risk into absolute risk for a meaningful time period	Limitations acknowledges this was not done due to small sample
Other analyses	17	Report other analyses done — e.g., analyses of subgroups and interactions, and sensitivity analyses	Firth penalised regression (S4 Fig, S7 Table); BH-FDR correction (Results, ECG section); NLP agreement analysis (Table 2, S2–S4 Tables); beat-by-beat permutation test (Fig 5)
**Discussion**			
Key results	18	Summarise key results with reference to study objectives	Discussion, paras. 1–6
Limitations	19	Discuss limitations of the study, taking into account sources of potential bias or imprecision. Discuss both direction and magnitude of any potential bias	Discussion, Limitations and strengths: 8 limitations enumerated (i–viii)
Interpretation	20	Give a cautious overall interpretation of results considering objectives, limitations, multiplicity of analyses, results from similar studies, and other relevant evidence	Discussion, Methodological considerations (6 sub-paragraphs); Conclusion: "preliminary and hypothesis-generating"
Generalisability	21	Discuss the generalisability (external validity) of the study results	Limitations (vii): single centre, 6-month window, Colombian population; Future research: multicentre ≥500 patients
**Other information**			
Funding	22	Give the source of funding and the role of the funders for the present study and, if applicable, for the original study on which the present article is based	Financial disclosure: Minciencias Colombia, call 895-2021, project code 563-2021; funders had no role in study design, data collection, analysis, or publication

Reference: von Elm E, Altman DG, Egger M, Pocock SJ, Gøtzsche PC, Vandenbroucke JP; STROBE Initiative. Strengthening the Reporting of Observational Studies in Epidemiology (STROBE): explanation and elaboration. *Ann Intern Med.* 2007;147(8):W163–W194.
