# Supplementary material for: Exploratory association between multimodal AI-derived digital biomarkers and in-hospital mortality in adult patients with pneumonia: A proof-of-concept study
Source: PLOS Digit Health. 2026 Apr 30;5(4):e0000960. doi: 10.1371/journal.pdig.0000960 (PMC13132438; doi:10.1371/journal.pdig.0000960)
Supplement: S2 Appendix — Three representative, fully anonymised Spanish clinical note excerpts with annotated IDSA/ATS criterion matches from the NLP pipeline. (PDF) [file pdig.0000960.s019.pdf]

S2 Appendix. Anonymised NLP Extraction Examples (Spanish Clinical Notes)

Exploratory multimodal artificial intelligence biomarker analysis for in-hospital mortality in adult patients with pneumonia: a proof-of-concept study

Three representative excerpts from anonymised Spanish clinical notes are presented below, illustrating how the NLP pipeline extracts modified IDSA/ATS severity criteria. Each example shows: (a) a verbatim excerpt from the patient's electronic health record, (b) the criteria detected by the regex engine, and (c) the resulting severity score. Patient identifiers correspond to the anonymised numeric codes used throughout the study; dates and institution names have been removed. All excerpts were processed through the NLP module running in Docker (Humath Curie NLP v2.0, exact-match mode with fuzzy threshold = 95%).

Example 1 — Patient 7035909: low severity (IDSA/ATS score: 1)

Input Clinical Note excerpt (Spanish)

Cirugía de Tórax
, 26 años

Diagnósticos:
– Neumonía necrotizante multilobar
– Empiema pleural derecho
– Fuga aérea

Cirugía
– POP VATS derecha, decorticación pulmonar, extracción de coágulos, lobectomía parcial LSD, lavado de cavidad. Dos drenajes pleurales

Antecedentes
– Patológicos: ¿cardiopatía? Refiere que hace 3 años le realizaron un cateterismo, desconoce resultado.
– Toxicológicos: tabaquismo y consumo de THC

TENDENCIAS ÚLTIMAS 24 HR
Sin fiebre
Episodios de bradicardia de 57
Episodios de hipotensíon de 85/54 88/56
Saturación normal

**Extracted age:**	26 years
**Major criteria detected:**	None
**Minor criteria detected:**	Multilobar (multilobar opacities on radiological imaging; score = 1)<br>Hipotensíon (hypotension requiring aggressive crystalloid resuscitation; score = 1)
**IDSA/ATS score:**	1 (age 0 + minor 2[†])
**Severe pneumonia (IDSA):**	No

[†]The NLP pipeline returned score = 1 rather than 2 because the age-based component contributes 0 points for patients <50 years. Both minor keywords (*multilobar*, *hipotensíon*) were matched via exact regex; however, "hipotensíon" here appears only

in the nursing trend section (blood pressure 85/54), not in a physician-authored severity assessment. This illustrates how context-free regex matching can inflate the criterion count: the word is present, but the clinical significance may be debatable.

Example 2 — Patient 7064240: severe pneumonia (IDSA/ATS score: 4)

Input Clinical Note excerpt (Spanish)

UCI Ronda
, 69 años

Diagnosticos
– Sepsis pulmonar
  * neumonía multilobar
  * derrame pleural paraneumonico
  * insuficiencia respiratoria aguda en cánula de alto flujo
  * hipoxemia moderada
– Aneurisma aorta abdominal
  ** Disección tipo Stanford B

Antecedentes:
– Patológicos: Hipertensión arterial, EPOC de novo, prediabetes, ACV hemorrágico en 2022
– Toxicológicos: Tabaquista pesada activa, 1 paquete desde los 10 años (IPA 59)

Soportes
Hemodinámico Noradrenalina (Suspendida 11am)
Ventilación mecánica volumen control 8.6/K PEEP 8 FIO2 40% FR 20

Episodios de hipotensíon de 71/39
Episodios de desaturación de 87 86 88

**Extracted age:**	69 years
**Major criteria detected:**	Ventilación mecánica (need for invasive mechanical ventilation; score = 2)<br>Noradrenalina → mapped to *shock séptico* (septic shock with need for vasopressors; score = 2)
**Minor criteria detected:**	Hipoxemia ($PaO_2/FiO_2 \leq 250$; score = 1)<br>Multilobar (multilobar opacities; score = 1)<br>Hipotensíon (hypotension requiring aggressive resuscitation; score = 1)
**IDSA/ATS score:**	4 (age 2 + major 4 + minor 3†)
**Severe pneumonia (IDSA):**	Yes (≥1 major criterion)

†The pipeline returned a total score of 4 because it caps the contribution of minor criteria when major criteria are present (IDSA/ATS rule: ≥1 major OR ≥3 minor = severe). Five keywords were matched by exact regex. Note that "noradrenalina" is listed as a synonym for the major criterion *shock séptico* in the keyword dictionary, correctly mapping a specific vasopressor drug name to the higher-level criterion. The note also describes "cánula de alto flujo" (high-flow nasal cannula), which does not trigger the mechanical ventilation criterion because the pipeline requires the explicit terms "ventilación mecánica" or "intubación".

Example 3 — Patient 7066720: moderate–high severity (IDSA/ATS score: 5)

Input Clinical Note excerpt (Spanish)

MEDICINA INTERNA
, 87 años

Diagnosticos:
– Neumonía adquirida en comunidad en tratamiento
  ** CURB-65: 3 puntos
– EPOC exacerbado
– Estenosis aórtica grave por disfunción de bioprótesis
– Fibrilación atrial de novo
– Lesión renal aguda KDIGO 2 en resolución
– Delirium

Antecedentes personales:
– Patológicos: Hipertensión arterial crónica, portador de bioprótesis valvular en posición aórtica, dislipidemia, EPOC GOLD 3 grupo E oxigenorrequiriente (15 horas), hipotiroidismo subclínico

S/ [...] con episodios de desorientación, inquietud y en ocasiones no reconoce a los familiares.

[...] gases arteriales con hipoxemia moderada, acidosis mixta [...]
taquipnea, no polipnea, no tirajes.
hemograma sin trombocitopenia, no leucocitosis [...]
sin hipotensíon, por el broncoespasmo recibe manejo [...]

**Extracted age:**	87 years
**Major criteria detected:**	None
**Minor criteria detected:**	Taquipnea (respiratory rate $\geq$30; score = 1)<br>Hipoxemia ($PaO_2/FiO_2 \leq 250$; score = 1)<br>Desorientación → mapped to *confusión* (confusion/disorientation; score = 1)<br>Trombocitopenia (platelet count <100 000; score = 1)<br>Hipotensíon (hypotension requiring aggressive resuscitation; score = 1)
**IDSA/ATS score:**	5 (age 2 + minor 5)
**Severe pneumonia (IDSA):**	Yes ($\geq$3 minor criteria)

This example exposes important false-positive limitations of context-free keyword matching. The note explicitly states "*sin* trombocitopenia" (without thrombocytopenia) and "sin hipotensíon" (without hypotension), yet the pipeline detects the keywords because it does not parse negation. In addition, "desorientación" was correctly mapped to the confusion criterion via the synonym list. The word "taquipnea" appears frequently in the clinical record in both positive and negative contexts (e.g., "no hay taquipnea"); only the presence of the token is counted. These false positives inflate the score from a likely true value of ∼3 to the reported 5, highlighting the need for negation-aware NLP in future iterations of the pipeline.

Summary of IDSA/ATS Criteria and NLP Keywords

Type	Criterion	Spanish keyword(s)	Score
Major	Invasive mechanical ventilation	ventilación mecánica, intubación	2
Major	Septic shock	shock séptico	2
Minor	Tachypnea ($\geq$30 breaths/min)	taquipnea	1
Minor	$PaO_2/FiO_2 \leq 250$	hipoxemia	1
Minor	Multilobar opacities	multilobar, bilateral, neumonía bilateral	1
Minor	Confusion	confusión	1
Minor	Uremia (BUN $\geq$20)	uremia	1
Minor	Leukopenia (WBC $<$4000)	leucopenia	1
Minor	Hypotension	hipotensión	1

Note: An age-based score component (0–2 points based on age thresholds) is added from structured EHR fields, not from the free-text extraction.
