# Supplementary material for: Exploratory association between multimodal AI-derived digital biomarkers and in-hospital mortality in adult patients with pneumonia: A proof-of-concept study
Source: PLOS Digit Health. 2026 Apr 30;5(4):e0000960. doi: 10.1371/journal.pdig.0000960 (PMC13132438; doi:10.1371/journal.pdig.0000960)
Supplement: Attachment — Submitted filename: Major concerns review comments.pdf [file pdig.0000960.s020.pdf]

Major concerns

1. CXR biomarker validation absent. You describe DeepLabV3 segmentation + ResNet-18 + CAM and a "quadrant-based lung injury quantification score" (p.25 l.428–433), but provide no internal test metrics (e.g., Dice for segmentation; AUC/F1 for classification; calibration for probabilities) on held-out data, nor external validation. This undercuts confidence in the "compromise ratio" associations.
2. Anatomical/labeling inconsistency driving a protective effect. The text states: "higher compromise ratio specifically in the right bottom portion of the left lung … associated with a significantly lower risk of mortality (OR 0.60…)" (p.13 l.179–181). This label is inconsistent (left lung cannot have a "right bottom portion") and likely originates from quadrant indexing or cardiac-silhouette overlap in CAM.
3. NLP evaluation with zero false positives. The manuscript states "There were no instances of false positives…" (p.20 l.268–272), despite numerous false negatives across criteria. This is implausible and suggests counting or denominator issues in how FP were defined.
4. ECG digitization not technically validated. ECGs were digitized from PDFs and processed with NeuroKit2 (p.27 l.445–448), but there is no benchmarking against original digital ECGs (e.g., Bland–Altman), no explicit sampling rate, filters, artifact handling, or lead specification.
5. Statistical fragility & multiplicity. Key signals (e.g., Klebsiella OR 18.38; 95% CI 1.80–187.75, p.13 l.172–176) have very wide CIs; only univariate/bivariate analyses are reported (p.8 l.79–80) despite many features. There is no multiple-testing correction or penalized modeling suitable for 19 events.
6. Outcome ascertainment not fully documented. Mortality was determined from EHRs and "confirmed through national registries" (p.2 l.18–19; p.27 l.449–450), but the registry name, linkage fields, and capture completeness are not described.
7. Selection bias from excluding DNI/RTI refusal. "We excluded patients who refused intubation…" (p.8 l.69–71; also p.2 l.14). This may bias mortality associations.
8. Data availability non-compliance. "Fully anonymized datasets might be available upon request." (p.5 l.1200–1220). PLOS policy requires open availability.
9. Reporting/presentation issues.
    - Keywords: duplicated comma ("Mort ality Prediction,, Digital Biomarkers") (p.8 l.90–92).
    - Author block formatting anomalies, e.g., "JoseJulian" (p.1 l.25).
    - Inconsistent stats labels: "Mean antibiotic duration (days), median [Q1,Q3]" (p.13 l.1–3).
    - Using "Median: 0.252±0.175 mV" mixes median with SD (p.15 l.228–231; p.16 l.232–233).

Specific questions (27 items; each cites where the issue appears or where clarification is needed)

1. CXR performance: What are segmentation (e.g., Dice) and classification (AUC/F1) metrics on a held-out internal set and, ideally, an external set? Please include calibration. (CXR pipeline described p.25 l.428–433.)
2. Compromise ratio derivation: How exactly are CAM maps converted to a numeric "compromise ratio"? Thresholding method, normalization, and inter-image standardization? (p.25 l.431–433; see also p.13 l.175–181 where it's analyzed.)
3. Quadrant mapping: Provide the lung-region schema and correct the "right bottom portion of the left lung" label; show an overlay figure with quadrants on a template CXR. (p.13 l.179–181.)
4. Sensitivity to cardiac silhouette/diaphragm: After fixing quadrant mapping, does the protective lower-lobe signal persist? Please re-run analyses excluding pixels overlapping the heart/mediastinum. (p.13 l.179–181.)
5. NLP FP definition: How were false positives defined for each IDSA/ATS criterion? Why are they recorded as zero? Please provide per-criterion confusion matrices. (p.20 l.268–272.)
6. Inter-rater reliability: Report Cohen's kappa (with CIs) for internist–internist and internist–AI agreement (you reference a kappa test, p.27 l.457–460). Please include exact values.
7. Source of demographics: Was age (and other demographics) pulled from structured fields or extracted by NLP? If NLP, please quantify extraction accuracy. (NLP pipeline p.27 l.451–458.)
8. ECG digitization parameters: Provide sampling rate after digitization, lead(s) used, and details on filtering, baseline wander removal, and artifact handling. (p.27 l.445–448.)
9. Technical validation vs. ground truth: Where possible, compare digitized PDFs to original digital ECG files (Bland–Altman, correlation) to validate amplitudes and HRV metrics. (p.27 l.445–448.)
10. Amplitude units/statistics: ECG amplitude results sometimes use "Median ± SD" (e.g., QRS and ST segments p.15 l.228–231; p.16 l.232–233). Please report either mean ± SD or median [IQR], consistently, and clarify units (mV).
11. Lead reporting: Which leads produced the amplitude features (I, II, V5, etc.)? If aggregated, how were amplitudes combined across leads? (ECG results p.14 l.190–198.)
12. Missing data handling: Beyond the >20% rule for exclusion (p.8 l.69–71), how were remaining missing values handled (complete-case vs. imputation)?
13. Mortality registry: Name the national registry used, linkage fields (ID, DOB), time lag, and capture completeness. (p.2 l.18–19; p.27 l.449–450.)
14. Events-per-variable: With 19 deaths (p.13 l.170–174), how many predictors did you consider per model? Please consider Firth or penalized logistic regression.

15. Multiple testing: Apply false discovery rate (e.g., Benjamini–Hochberg) across ECG/CXR/NLP features and indicate which associations persist.
16. Calibration & utility: If clinical translation is intended, add calibration plots and decision-curve analysis.
17. Klebsiella source: Did "principal diagnosis of Klebsiella pneumoniae" come from microbiology results or NLP of text? Please detail extraction rules and confirmation. (p.13 l.172–176.)
18. Time alignment: What were the time deltas between the first CXR, first ECG, and first clinical note relative to admission? (Methods overview p.8 l.66–74.)
19. Modalities flow diagram: Provide a STROBE-style flow showing how many patients had usable ECGs, CXRs, and notes.
20. Selection bias sensitivity: Repeat key analyses excluding patients who refused intubation (DNI/RTI) to assess selection effects. (p.8 l.69–71; p.2 l.14.)
21. Standardization details: For "per SD" ORs (e.g., ATS/IDSA severity, p.13 l.177–178), specify the population used to compute mean/SD and confirm z-scoring details.
22. Table label fix: Correct "Mean antibiotic duration (days), median [Q1,Q3]" (p.13 l.1–3) and harmonize statistics throughout.
23. Abstract grammar: Fix "validated internally data sources" (p.2 l.5–8) for grammar/clarity.
24. Keywords punctuation: Repair the duplicated comma in "Mort ality Prediction,, Digital Biomarkers." (p.8 l.90–92.) Also standardize capitalization/spacing across keywords.
25. Author block formatting: Correct fused name "JoseJulian" to "Jose Julian" and standardize degrees/punctuation (p.1 l.25).
26. Model training data leakage: Confirm that CXR fine-tuning (CES 2020 images; p.25 l.423–426) did not include images from the target hospital/period (2024 cohort) to avoid leakage.
27. Clarify registration header: The "Registration" header near the abstract lists IRB/funding details (p.8 l.81–87). If there was a pre-registered protocol/analysis plan, cite it; otherwise, relabel this section appropriately.

Actionable recommendations (for a major-revision resubmission)

- Open data & code: Replace "upon request" with compliant open sharing. Deposit de-identified data (or a shareable subset) and full pipelines (CXR/NLP/ECG) to a public repository with DOIs and include accession links in the manuscript. (p.5 l.1200–1220.)
- Validate AI components:
    - CXR: Report segmentation and classification metrics on held-out internal data; if feasible, add an external test. Provide calibration and error analysis. (p.25 l.428–433.)

- NLP: Publish per-criterion confusion matrices, prevalence, sensitivity/specificity, PPV/NPV, and kappa; address the current "no false positives" artifact. (p.20 l.268–272; p.27 l.457–460.)
- ECG: Add a technical validation against original digital ECGs; document sampling rate, filters, leads, and artifact handling. (p.27 l.445–448.)

- Fix the lung-region analysis: Correct quadrant labels, exclude cardiac silhouette overlap, and re-run regional associations. Explicitly revisit the "protective left-lower region" result. (p.13 l.179–181.)
- Strengthen statistics: Pre-specify a reduced feature set, apply multiplicity correction, and use penalized or Firth logistic regression with bootstrap CIs suitable for 19 events. Clearly report events-per-variable. (Deaths p.13 l.170–174; univariate/bivariate design p.8 l.79–80.)
- Document outcomes & selection: Describe the mortality registry linkage in detail and conduct sensitivity analyses excluding patients who refused intubation. (p.2 l.14; p.2 l.18–19; p.27 l.449–450.)
- Reporting polish:
  - Harmonize statistics (mean ± SD vs. median [IQR]) and fix mislabeled table entries (p.13 l.1–3).
  - Correct amplitude reporting that currently mixes "Median ± SD" (p.15 l.228–231; p.16 l.232–233).
  - Repair grammar in the abstract (p.2 l.5–8), duplicated comma in keywords (p.8 l.90–92), and author-block formatting (p.1 l.25).
  - Remove submission-system boilerplate and stray instruction lines (visible on p.5 and elsewhere).

Final decision

Decision: Reject (major revisions invited). The study is promising and clearly presented as exploratory, but it requires (i) transparent validation of the AI pipelines, (ii) correction of the lung-region labeling and re-analysis, (iii) more robust statistical handling given 19 events, and (iv) policy-compliant data/code availability before it can be considered for publication.
