# Supplementary material for: Exploratory association between multimodal AI-derived digital biomarkers and in-hospital mortality in adult patients with pneumonia: A proof-of-concept study
Source: PLOS Digit Health. 2026 Apr 30;5(4):e0000960. doi: 10.1371/journal.pdig.0000960 (PMC13132438; doi:10.1371/journal.pdig.0000960)
Supplement: Attachment — Submitted filename: rebuttal_letter.pdf [file pdig.0000960.s022.pdf]

Rebuttal Letter

Manuscript ID: PDIG-D-25-00464
Title: Exploratory association between multimodal AI-derived digital biomarkers and in-hospital mortality in adult patients with pneumonia: a proof-of-concept study
Journal: PLOS Digital Health
Revision type: Major Revision
Date: March 31, 2026

Dear Editor and Reviewers,

We thank the Editor and all eight Reviewers for their thorough, constructive evaluation of our manuscript. The comments have substantially improved the scientific rigour, transparency, and reproducibility of our work. Below we provide a point-by-point response to every comment, organised by reviewer. For each item we indicate: (i) the original comment (abbreviated), (ii) our response, and (iii) the specific correction made in the revised manuscript or supplementary files. Line numbers refer to the revised manuscript unless stated otherwise.

Notation. Throughout the tables below we use the following abbreviations in the *Change* column:

- **MS** = Main manuscript text
- **SI** = Supporting Information file(s)
- **RO** = Rebuttal only (no manuscript change required)

A marked-up version of the manuscript showing all changes (using `latexdiff`) is provided as a separate file (`manuscript_diff.pdf`).

Response to Journal Requirements (Editor)

#	Comment	Response	Manuscript Correction
JR-1	Competing Interests statement required.	Added.	**MS:** Added *Competing interests* section: "The authors have declared that no competing interests exist."
JR-2	Financial Disclosure — full sentences, author initials, funder role.	Added with all required details.	**MS:** Added *Financial disclosure* section with Minciencias call 895-2021, project code 563-2021, author initials, and funder-role statement: "The funders had no role in study design, data collection and analysis, decision to publish, or preparation of the manuscript."
JR-3	Ethics Statement at start of Methods (IRB name, approval number, consent status).	Added as the first subsection of Materials and Methods.	**MS:** *Ethics Statement* subsection (L636): Comité de Ética, Hospital Alma Máter de Antioquia; approval IN82-2021; retrospective waiver of informed consent.
JR-4	Author Summary (150–200 words, first person, between Abstract and Introduction).	Written and inserted.	**MS:** *Author Summary* section (∼170 words, first-person voice) placed between Abstract and Introduction.
JR-5	Remove figures from manuscript body; submit each as separate TIFF.	All seven main figures exported as TIFF and uploaded individually. Figure captions remain inline per PLOS style.	**MS:** Figures removed from body; Fig 1–Fig 7 TIFFs uploaded. Captions retained inline after first citation.
JR-6	Upload S2 Table (kappa) as Supporting Information file.	Uploaded as part of the combined workbook `S_Tables_S1_S9.xlsx`. Individual sheet names match S-item numbers.	**SI:** S2 Table included in `S_Tables_S1_S9.xlsx` (tab "S2").
JR-7	Add Supporting Information captions at end of manuscript.	Done.	**MS:** *Supporting Information* section added after References listing all S-item legends (S1–S5 Fig, S1–S9 Table, S1–S2 Appendix, S6 Table).
JR-8	Data availability — deposit de-identified feature tables publicly.	De-identified feature tables (CXR ratios, NLP indicators, ECG/HRV metrics), anonymised raw clinical data, and analysis code have been deposited on Zenodo (DOI: 10.5281/zenodo.19361102). The Data Availability Statement in the manuscript has been updated accordingly.	**MS:** Data Availability Statement added with Zenodo DOI (`https://doi.org/10.5281/zenodo.19361102`).

#	Comment	Response	Manuscript Correction
JR-9	Intubation exclusion justification in Ethics Statement.	The separate justification paragraph has been removed from the Ethics Statement. Because the study is an exploratory analysis of associations with in-hospital mortality—not an intubation-outcome study—patients who refused intubation were excluded as their treatment-limiting decisions directly alter the mortality endpoint under investigation. The exclusion criterion itself remains documented in the eligibility criteria (Materials and Methods).	**MS:** Intubation exclusion paragraph removed from Ethics Statement; exclusion criterion retained in study design section.

Response to Reviewer #1 (19 Comments)

#	Comment	Response	Manuscript Correction
R1-1	"Synergistic value" claim unsupported → reframe as exploratory or fit multivariable model.	We agree. The manuscript has been comprehensively reframed as an *exploratory, proof-of-concept* study. The title now includes "Exploratory" and "proof-of-concept." Claims of synergistic or predictive value have been removed throughout.	**MS:** Title, Abstract, Introduction (final paragraph), Discussion, and Conclusion revised to use exploratory language consistently.
R1-2	Bivariate only; add joint multivariable model (CXR+NLP+ECG) with ΔAUC/ΔBrier.	A Firth penalised logistic regression with three predictors (one per modality) was fitted as a sensitivity analysis ($n = 121$, 19 events, EPV≈4). Results: AUC = 0.756, Brier = 0.116. This is explicitly framed as exploratory and underpowered.	**MS:** Statistical Analysis and new "Exploratory multimodal model" paragraph in Results (L757). **SI:** S7 Table (Firth coefficients), S4 Fig (calibration plot).
R1-3	Multiple testing without correction; apply BH-FDR; add q-value column.	Benjamini–Hochberg FDR applied to all univariate tests. After tachycardia-corrected re-analysis (v4.0; 383 beats from 27 patients), no ECG feature survives FDR at $q < 0.05$; the strongest signals are RR-interval features ($q = 0.068$). Ultra-short HRV analysis and lead-by-lead comparisons provide complementary evidence: three HRV metrics (HR, RMSSD, SD1) reached $p < 0.05$, and 4/39 lead-level features survived FDR (T-wave amplitude in leads II, III, V5; ST-level in lead II strip).	**MS:** Results updated with v4.0 q-values; HRV and lead-by-lead paragraphs added. **SI:** S6 Fig (HRV boxplots), S7 Fig (lead-by-lead).
R1-4	Anatomical mislabel ("right bottom portion of left lung"); OR biologically counterintuitive.	Corrected. The region was the right lower lung zone (radiographic left lower zone). A footnote on the cardiac silhouette artefact has been added.	**MS:** Results CXR section (L362) corrected; Discussion paragraph added acknowledging potential CAM artefact from cardiac silhouette.
R1-5	Wide CIs (OR 18.38, 24.33) → penalised models, VIF, splines for non-linear effects.	Wide CIs are acknowledged as a direct consequence of $n = 19$ events. Firth penalised ORs are reported in S7 Table. VIF computed in S9 Table (all VIF < 3). Restricted cubic splines were not fitted owing to insufficient degrees of freedom.	**MS:** Statistical Analysis paragraph added (L770); Limitation expanded. **SI:** S9 Table (VIF).
R1-6	CXR pipeline: held-out Dice/IoU, AUC, CAM threshold definition.	Validated against 106-image radiologist ground truth using LOOCV: Dice = 0.286±0.199, AUROC = 0.613±0.223, $T^* = 0.20$.	**MS:** Methods CXR subsection (L670) now reports Dice, AUROC, and T^* with LOOCV methodology.

#	Comment	Response	Manuscript Correction
R1-7	ECG technical details: sampling rate, filters, leads, R-peak QC, rhythm exclusions.	All details added. ECG analysis upgraded to v4.0 with tachycardia-overlap correction, multi-lead extraction, and ultra-short HRV from standardised lead II (10-second segment). Beat counts updated to 326 survivor / 57 non-survivor beats (383 total) after quality filtering. HRV literature comparison added to Discussion citing Junior et al. 2019, de Castilho et al. 2018, Papaioannou et al. 2009, and Nussinovitch et al. 2011/2012 on 10-second HRV reliability (5 new references added).	**MS:** ECG subsection (L680): 500 Hz interpolated from pixel density, lead II, 0.5–40 Hz bandpass (NeuroKit2), Pan-Tompkins R-peak detection, AF/paced exclusion documented in Fig 1. Results: HRV and lead-by-lead paragraphs added. Discussion: two new paragraphs comparing HRV findings with sepsis literature and discussing 10-second ECG reliability. **SI:** S6 Fig (HRV boxplots), S7 Fig (lead-by-lead analysis).
R1-8	Report kappa in main text; per-criterion F1; clarify V1 vs V2.	Kappa values inserted inline with 95 % CIs from bootstrap.	**MS:** Results NLP (L468): $\kappa_{\text{inter}} = 0.408$ [0.223–0.592], $\kappa_{\text{V1}} = 0.510$ [0.336–0.684], $\kappa_{\text{V2}} = 0.455$ [0.275–0.635]. V1 = dictionary-only; V2 = dictionary+context window. **SI:** S3 Table (per-criterion F1).
R1-9	FN impact on ATS/IDSA score distribution; measurement-error analysis.	Computed: NLP false negatives would reclassify ∼15–20 % of patients to a lower severity tier under ATS/IDSA scoring. Added as sensitivity paragraph.	**MS:** Results NLP section (new paragraph); Methodological Considerations in Discussion expanded.
R1-10	STROBE flow diagram + missingness table + included vs. excluded comparison.	Fig 1 is now a STROBE-compliant flow diagram. Missingness data in S1 Table.	**MS:** Fig 1 (L264); exclusion criteria paragraph in Methods. **SI:** S1 Table (missingness by variable).
R1-11	Time-to-event sensitivity (Cox); report LOS by outcome, time-to-death.	Time-to-death for non-survivors: median 12.0 days [IQR 5.0–21.5]. Cox regression was not performed owing to insufficient events ($n = 19$); this rationale is discussed explicitly.	**MS:** Results Cohort section (new sentence); Discussion "Time-to-event considerations" paragraph added.
R1-12	Data availability — Zenodo/GitHub deposit.	De-identified feature tables, anonymised raw clinical data, and analysis code deposited on Zenodo (DOI: 10.5281/zenodo.19361102). See JR-8.	**MS:** Data Availability Statement added with Zenodo DOI.
R1-13	Duplicate citation [11],[12],[11].	Fixed.	**MS:** Duplicate removed (L23). Reference list audited.
R1-14	Version placeholder in front matter.	Removed.	**MS:** No versioning artefacts remain.

#	Comment	Response	Manuscript Correction
R1-15	CXR segmentation metrics on held-out set; train/val/test splits.	Described. Pre-training datasets: CheXpert ($N = 224{,}316$), NIH ChestX-ray14 ($N = 112{,}120$). Fine-tuning: 395 images Clínica CES. LOOCV for T^*.	**MS:** Methods CXR (L654): dataset provenance and split strategy described.
R1-16	Consistent anatomical scheme for lung zones.	Lung zones are now defined as right upper, right lower, left upper, and left lower using radiographic convention. A schematic has been added.	**MS:** Terminology unified throughout. **SI:** S5 Fig (lung zone schematic with right/left zone labels).
R1-17	ECG amplitude units (mV), lead specification, baseline definition.	Added.	**MS:** (L697): "All ECG amplitudes are reported in millivolts (mV); the isoelectric baseline was estimated as the TP-segment mean." Lead II specified.
R1-18	19 events — EPV statement, cap predictors, optimism-corrected performance.	EPV ≈ 4 stated in Abstract and Limitations. Firth model capped at 3 predictors.	**MS:** Abstract (L169); Statistical Analysis; Limitations (L570).
R1-19	Broader cross-domain citations (ECG transformers, paleontology, geoscience, engineering).	A cross-domain perspective paragraph citing the ECG transformer survey (Artif. Intell. Rev. 2025) has been added. The paleontology, geoscience, and engineering analogies were removed in the final revision as they did not directly address the clinical informatics scope; the paragraph now focuses exclusively on transformer-based ECG architectures relevant to LMIC settings.	**MS:** Discussion "Cross-domain perspective" paragraph revised. **RO:** Non-clinical domain analogies removed for conciseness.

Response to Reviewer #2 (1 Comment)

#	Comment	Response	Manuscript Correction
R2-1	Supports the work; asks for future trials and higher accuracy tools.	We thank Reviewer #2 for the positive assessment. The Discussion now includes a detailed Future Directions paragraph specifying: (i) a planned multicentre prospective study ($\geq$500 patients), (ii) architecture comparison (ResNet-18 vs. ViT/CoCa), (iii) sex- and age-stratified sub-analyses, and (iv) an NLP upgrade pathway (transformer-based Spanish models).	**MS:** Discussion "Future directions" paragraph expanded.

Response to Reviewer #3 (8 Major Clusters)

#	Comment	Response	Manuscript Correction
R3-1	CXR pipeline: Dice, AUC/F1, calibration.	Addressed (same as R1-6). Dice = 0.286±0.199, AUROC = 0.613±0.223, $T^* = 0.20$ via LOOCV.	**MS:** Methods CXR subsection (L670).
R3-2	"Compromise ratio" mathematically undefined; quadrant labels unstable.	The compromise ratio (CR) is now formally defined: CR = (CAM-activated pixels / total zone pixels) × 100, where activation $\geq T^* = 0.20$. The total compromise ratio (TCR) is defined as the area-weighted mean across zones.	**MS:** Methods CXR subsection: equation environment added. Terminology unified from "quadrant" to "zone."
R3-3	NLP zero FP not credible; confusion matrices; kappa with 95 % CIs.	The regex-based NLP design matches only explicit keyword patterns; therefore, false positives are structurally impossible when the keyword is absent (the system errs toward false negatives by design). Kappa 95 % CIs are now reported inline (see R1-8).	**MS:** NLP results updated with CIs. **SI:** S2 Table (confusion matrices), S3 Table (per-criterion F1).
R3-4	ECG: Bland-Altman vs native digital ECGs.	Native digital ECG recordings (machine-output XML or SCP-ECG) were **not available** for this retrospective cohort; all ECGs were archived as printed paper reports and subsequently digitised. In the absence of paired digital reference traces, a classical Bland-Altman comparison against machine output cannot be performed. We acknowledge this explicitly as a limitation of the digitisation approach in a resource-limited LMIC setting.	**MS:** Methods ECG (L698): explicit statement of unavailability; Limitations item expanded. **RO:** Bland-Altman declined with rationale.
R3-5	BH-FDR, EPV, Firth penalised regression.	All addressed (see R1-2, R1-3, R1-18).	**MS:** Statistical Analysis; Results tables updated with q-values; Firth model in S7 Table.
R3-6	STROBE diagram; outcome documentation; modality time alignment.	STROBE flow diagram added as Fig 1 (see R1-10). Time alignment statement added.	**MS:** Methods: "All three modalities were obtained within ±24 hours of hospital admission."
R3-7	Open data and code sharing.	De-identified feature tables, anonymised raw clinical data, and analysis code deposited on Zenodo (DOI: 10.5281/zenodo.19361102). See JR-8.	**MS:** Data Availability Statement added with Zenodo DOI.
R3-8	Presentation: units, median[IQR] vs mean±SD, grammar.	All continuous variables now reported as median [Q1, Q3]. Units standardised. Grammar reviewed throughout.	**MS:** Table 1 and all Results sections updated.

#	Comment	Response	Manuscript Correction

Response to Reviewer #4 (7 Major + 4 Minor)

#	Comment	Response	Manuscript Correction
R4-1	Modern architectures (transformers, attention) vs basic ResNet-18.	ResNet-18 was chosen for three reasons: (i) established precedent for CXR tasks (CheXNet, Rajpurkar et al. 2017), (ii) parameter efficiency enabling CPU-only deployment in resource-limited settings ($\sim$11 M parameters vs >86 M for ViT-Base), and (iii) inference in <200 ms on a standard CPU. A comparison with larger architectures is planned as future work.	**MS:** Methods CXR paragraph; Discussion "Methodological considerations" paragraph. ViT/CoCa cited.
R4-2	No true multimodal fusion — only univariate analysis.	We agree that no formal fusion was performed. The study is reframed as an *exploratory association study.* The Firth logistic model is presented as a proof-of-concept sensitivity analysis, not a validated predictor.	**MS:** Title, Abstract, and Discussion revised consistently.
R4-3	$N = 121$ underpowered; EPV violation ($n = 19$ events).	Acknowledged. EPV $\approx$ 4 stated explicitly. The study is declared hypothesis-generating throughout.	**MS:** Abstract, Statistical Analysis, Limitations.
R4-4	Wide CIs (Klebsiella OR 18.38, HR OR 24.33) clinically irrelevant.	Acknowledged as a limitation of small n; Firth-corrected ORs provided in S7 Table.	**MS:** Discussion; **SI:** S7 Table.
R4-5	No comparison with CURB-65/PSI benchmarks.	The study used the IDSA/ATS severity score as the clinical benchmark. CURB-65 requires variables (BUN, respiratory rate, systolic BP, clinical confusion assessment) that were not systematically available in the retrospective dataset. IDSA/ATS is the guideline-recommended severity stratification tool and serves the equivalent benchmarking role.	**MS:** Discussion "Methodological considerations" paragraph added with IDSA/ATS vs CURB-65 rationale. **RO:** CURB-65 retrospective calculation declined with rationale.
R4-6	No computational requirements or EHR integration discussion.	Added.	**MS:** Discussion: full pipeline runs in 5–10 s per patient on a standard CPU. EHR integration pathway described (FHIR-compatible outputs).
R4-7	Statistical limitations (univariate only, no CV, no FDR).	Addressed by R1-2, R1-3, R1-18 above.	See R1-2, R1-3, R1-18.

#	Comment	Response	Manuscript Correction
R4-m1	Protective OR for lower lung compromise contradicts clinical intuition.	Footnote added acknowledging the cardiac silhouette artefact as a potential confounder for the lower-left zone CAM activation; causal language removed.	**MS:** Results CXR and Discussion.
R4-m2	20 % missing data threshold arbitrary.	Justified with citation: Sterne et al. 2009 (BMJ) recommending ≤20 % threshold for complete-case analysis with EPV constraints.	**MS:** Statistical Analysis (citation added).
R4-m3	Image quality standardisation not discussed.	Inclusion criteria added.	**MS:** Methods CXR: "Inclusion required AP or PA projection, absence of significant rotation or truncation, and sufficient contrast for anatomical landmark identification."
R4-m4	ECG digitisation accuracy not validated.	Same as R3-4: no native digital ECGs exist for this retrospective cohort. This is acknowledged as an LMIC limitation.	**MS:** Limitations expanded. **RO:** See R3-4 rationale.

Response to Reviewer #5 (4 Items)

#	Comment	Response	Manuscript Correction
R5-1	Abstract overloaded with methodological details.	Rewritten as continuous prose (∼220 words) focusing on key findings and the exploratory nature of the study. Sub-headings removed per PLOS style.	**MS:** Abstract entirely rewritten.
R5-2	No related work review; no comparison with existing approaches.	A "Related Work" subsection has been added to the Introduction covering prior CXR AI models for pneumonia, multimodal fusion approaches, ECG digitisation studies, and Spanish-language clinical NLP.	**MS:** Introduction, new paragraphs (∼0.5 page).
R5-3	Methods: explicit distinction between CXR (pretraining+fine-tuning) vs ECG/NLP (feature extraction for statistics).	An explicit statement has been added.	**MS:** Data Sources subsection: "Deep learning pretraining and fine-tuning were applied only to the CXR module; the ECG and NLP modules used traditional feature extraction for downstream statistical analysis."
R5-4	CXR pretraining details: patient counts, epochs, hyperparameters.	Full hyperparameter table recovered and added to Supporting Information.	**SI:** S6 Table: ResNet-18, 300 epochs, AdamW (lr = 0.005, $\beta_1 = 0.9$, $\beta_2 = 0.999$, wd = 0.05), batch size 512, cosine annealing with warm restarts, input 224×224, 3 classes (normal/consolidation/other), knowledge distillation ($\alpha = 0.5$, $T = 4.0$, DenseNet-121 teacher).

Response to Reviewer #6 (2 Items)

#	Comment	Response	Manuscript Correction
R6-1	CXR model performance evaluation before association analysis.	Done (same as R1-6). Dice and AUROC reported in Methods prior to any association results.	**MS:** Methods CXR (L670).
R6-2	NLP: unclear model; more examples of reports and extracted scores.	The NLP module is a deterministic regex-based system (not a machine learning model); this has been clarified in the text. Anonymised examples of Spanish clinical notes with extracted ATS/IDSA criteria annotations are provided in S2 Appendix.	**MS:** NLP subsection clarified. **SI:** S2 Appendix (`S2_Appendix_NLP_Examples.pdf`) with annotated examples.

Response to Reviewer #7 (Multiple Clusters)

#	Comment	Response	Manuscript Correction
R7-1	Use impersonal language; remove "we" pronouns.	Audited. First-person pronouns are now limited to the Author Summary (required by PLOS) and one standard "to our knowledge" phrase in the Introduction. Results and Discussion use impersonal constructions.	**MS:** Global edit throughout Results and Discussion.
R7-2	Novelty not stated clearly in abstract and main text.	Explicit novelty claim added.	**MS:** Introduction: "This study is, to our knowledge, the first to integrate automatic CXR consolidation quantification, Spanish-language NLP-based clinical severity scoring, and quantitative ECG analysis from digitised paper archives within a single LMIC cohort."
R7-3	NLP regex limitations not stated (abbreviations, negation, cultural variation, shallow semantics).	Expanded.	**MS:** Limitations item (vi): abbreviation ambiguity, negation resolution, cultural variation, and regex vs ML rationale discussed.
R7-4	NLP preprocessing steps not detailed.	Added.	**MS:** NLP Pipeline subsection (L709): lowercasing, accent normalisation, non-semantic token removal, pattern-matching logic described.
R7-5	Class imbalance handling.	Statement added.	**MS:** Statistical Analysis: "Class imbalance ($n_{\text{dead}} = 19$, $n_{\text{alive}} = 102$) was addressed by using exact tests (Fisher's exact) and reporting odds ratios with 95 % CI rather than accuracy-based metrics." King & Zeng 2001 cited.
R7-6	ECG preprocessing beyond digitisation.	Described (same as R1-7).	**MS:** ECG subsection: bandpass 0.5–40 Hz, Pan-Tompkins R-peak detection, artefact exclusion (RR 300–2000 ms).
R7-7	Why only CAM? Justify vs alternatives (Grad-CAM++, SHAP, LIME).	CAM was selected for its computational efficiency and direct compatibility with the ResNet-18 global average pooling architecture. Grad-CAM, Grad-CAM++, SHAP, and LIME are cited as alternatives for future work.	**MS:** Methods CXR paragraph; Discussion "Methodological considerations."

#	Comment	Response	Manuscript Correction
R7-8	Why ResNet-18? Justify vs modern architectures (CoCa, ViT, etc.).	Same as R4-1.	**MS:** Methods; Discussion.
R7-9	Temporal and selection bias.	Both addressed.	**MS:** Limitations item (vii): single-centre, 6-month window, demographics constrained; selection bias from DNI/DNR exclusion justified.
R7-m	Minor: L222 title unreadable; L173 bacteria not italicised; L304 missing period; COPD definition; intubation exclusion; sex vs gender.	All corrected. Intubation exclusion criterion retained in study design (Methods, eligibility criteria) but the separate Ethics Statement paragraph was removed; the rationale is that this is an exploratory mortality analysis and patients who refused intubation have a fundamentally different outcome trajectory that would confound the primary mortality endpoint.	**MS:** *Klebsiella pneumoniae* italicised; COPD spelled out on first use; intubation exclusion justification paragraph removed from Ethics Statement (rationale provided in eligibility criteria); "sex" vs "gender" audited per SAGER guidelines (Limitation viii).

Response to Reviewer #8 (5 Thematic Sections)

#	Comment	Response	Manuscript Correction
R8-1	Reframe as hypothesis-generating.	Done consistently throughout (see R1-1).	**MS:** Title, Abstract, Results, Discussion, and Conclusion revised.
R8-2	Validated models, benchmarks, calibration, multiplicity control.	Firth model with AUC = 0.756, Brier = 0.116 added as sensitivity analysis. BH-FDR applied. CURB-65 justification added (see R4-5).	**MS:** Statistical Analysis; Results. **SI:** S7 Table, S4 Fig.
R8-3	Sensitivity analyses for methodological uncertainties.	Firth sensitivity analysis performed. CXR projection-type and saliency robustness acknowledged as limitations.	**MS:** Limitations item (v). **SI:** S7 Table.
R8-4	Consent framework; fairness/subgroup analysis by sex/age.	Consent waiver stated in Ethics. Sex-stratified mortality (13.7 % female, 17.1 % male) and age-stratified mortality ($\leq$65: 12.3 %, >65: 19.6 %) added in Results. SAGER audit noted.	**MS:** Ethics Statement; Results Cohort section; Limitations item (viii).
R8-5	Data availability (Zenodo/code deposit).	De-identified feature tables, anonymised raw clinical data, and analysis code deposited on Zenodo (DOI: 10.5281/zenodo.19361102). See JR-8.	**MS:** Data Availability Statement added with Zenodo DOI.

Additional Author-Initiated Revisions (R2)

The following changes were made by the authors during the R2 revision to improve accuracy, conciseness, and transparency. None alter the study's findings or conclusions.

#	Change	Rationale	Location
A-1	Removed palaeontology and engineering analogies from the cross-domain perspective paragraph.	These analogies, while illustrative, are outside the clinical informatics scope and were flagged as tangential. The paragraph now focuses on transformer-based ECG architectures directly relevant to the study.	**MS:** Discussion, "Cross-domain perspective" paragraph.
A-2	Removed limitation item (v) regarding unarchived CXR AI training hyperparameters.	The full hyperparameter table (ResNet-18, 300 epochs, AdamW, etc.) has been recovered and documented in S6 Table, making this limitation obsolete.	**MS:** Limitations paragraph; remaining items renumbered.
A-3	Replaced "the study predates wide COVID-19 circulation" with "the study was conducted at a single centre during a six-month window (January–June 2024)".	The original phrasing was inaccurate—the study period (Jan–Jun 2024) postdates COVID-19 emergence. The revised text states the actual temporal and geographic scope without reference to COVID-19.	**MS:** Limitations, item (vii).
A-4	Added S8 Fig (CAM activation threshold LOOCV sweep) to Supporting Information.	Provides visual evidence of the threshold optimisation cited in the Methods ($T^* = 0.20$), enhancing reproducibility and transparency of the CXR pipeline.	**MS:** Methods (CXR pipeline), added "S8 Fig" reference. **SI:** New S8 Fig with caption.
A-5	Moved AF/paced rhythm exclusion documentation from S8 Table reference to Fig 1 (STROBE).	The manuscript previously cited "S8 Table" for AF/paced exclusion counts, but S8 Table actually contains ECG beat-level feature descriptives. The STROBE flow diagram is the appropriate location for exclusion criteria; none of the 27 ECG patients met AF/paced exclusion criteria. S8 Table description corrected to match actual content.	**MS:** Methods (ECG pipeline); Fig 1 caption updated. **Script:** STROBE E2 exclusion box updated.
A-6	Simplified NLP pipeline description by removing V1/V2 version distinction.	The manuscript now reports only the final pipeline configuration (dictionary-based regex with context-window rules) rather than describing the iterative development history, improving readability.	**MS:** Methods, NLP pipeline subsection.

Summary of Outstanding Items

The following items require actions external to the manuscript text and will be completed during the submission upload process:

1. **Zenodo data deposit** (JR-8, R1-12, R3-7, R8-5): **Completed.** De-identified feature tables, anonymised raw clinical data, and analysis code have been deposited on Zenodo (DOI: https://doi.org/10.5281/zenodo.19361102; https://zenodo.org/records/19361102). The Data Availability Statement in the manuscript has been updated with this DOI.
2. **PLOS submission system entries**: Competing Interests, Financial Disclosure, CRediT author contributions, and corresponding author ORCID will be entered via the online submission form.
3. **Striking image**: A 600 dpi CXR with CAM overlay will be uploaded as the striking image.

We trust that these revisions fully address all concerns raised by the Editor and Reviewers. We remain available for any further clarifications.

Respectfully,

Alejandro Hernández-Arango, on behalf of all authors.
